# Antireflective, photocatalytic, and superhydrophilic coating prepared by facile sparking process for photovoltaic panels

**DOI:** 10.1038/s41598-022-05733-7

**Published:** 2022-01-31

**Authors:** W. Thongsuwan, W. Sroila, T. Kumpika, E. Kantarak, P. Singjai

**Affiliations:** 1 Department of Physics and Materials Science, Faculty of Science, Chiang Mai University, Chiang Mai, 50200 Thailand; 2 Materials Research Centre, Faculty of Science, Chiang Mai University, Chiang Mai, 50200 Thailand; 3 Centre of Excellence in Materials Science and Technology, Chiang Mai University, Chiang Mai, Thailand

**Keywords:** Solar cells, Nanoparticles

## Abstract

Soiling of photovoltaic modules and the reflection of incident light from the solar panel glass reduces the efficiency and performance of solar panels; therefore, the glass should be improved to have antifouling properties. In this work, commercial solar panels were coated with sparked titanium films, and the antireflective, super-hydrophilic, and photocatalytic properties of the films were investigated. The reflectance, photocatalytic properties, and degradation of the organic pollutant methylene blue were determined using UV–Vis spectroscopy. The wetting properties were studied by measuring the water contact angle using an optical tensiometer. The outdoor power of the spark-discharged-titanium coated and uncoated PV panels was measured for 10 months at Chiang Mai, Thailand. It was found that conditions such as cloudiness, rainfall, and muddy stains significantly influenced the power difference (ΔP) between the coated and uncoated PV panels. The increase in ΔP was due to the improved dust removal from the super-hydrophilic surface of the coated panels. On a cloudy day, ΔP reached its highest value of 14.22%, which was anticipated to improve the anti-reflection property of the coated glass. The average ΔP was 6.62% over the entire experimental period.

## Introduction

To mitigate the harmful effects of burning fossil fuels on air quality and the global climate, renewable and sustainable energy sources are being given more attention. Solar power technologies offer interesting solution, but their power conversion efficiency (PCE) must be improved to ensure efficiency. Although solar photovoltaic panel cover glass is highly transparent, it has a natural reflectance in the visible wavelength range. An effective method to increase the effectiveness is to reduce the optical loss and natural reflectance via antireflection (AR) coatings.

Several methods to reduce the reflectance and enhance the efficiency of solar panels have been studied. Coating may be realized by both chemical and physical methods, such as sol–gel dip-coating^1^, spin coating^2^, nanoimprint lithography using sol–gel materials^3^, plasma surface oxidation^4^, RF sputtering^5–7^, and thermal evaporation^8^. In this work, we propose a simple and inexpensive sparking process to produce an AR film. This method uses simple equipment that can be operated in ambient conditions without a high-vacuum system. Furthermore, it does not generate toxic waste from chemical precursors or other chemical agents. The sparking process is a new synthesis method that can create porous nanostructured films with well-controlled thickness and uniform composition, as well as facilitate large-scale, one-step coating at a low cost^9–12^. It utilizes the sparking between two metal tips to form nanoparticulate films under atmospheric conditions so that an expensive vacuum system is not required. Therefore, it can be easily scaled up for commercial use. Furthermore, to the best of our knowledge, there have been no previous reports of the preparation of AR coatings for solar cell applications by the sparking method.

In addition to the reflectance of light from the glass cover, dust deposition on PV systems has become a serious problem, reducing the PV efficiency performance^13,14^. Dust has many sources, including pollution and wind. Manual and automated cleaning methods are recommended to reduce the settling of dust on solar panels. Manual methods require large quantities of water, whereas automated cleaning methods are a good solution for saving water. However, the high maintenance cost cannot be justified in comparison with the comparatively low advantages of dust reduction. Therefore, self-cleaning surfaces (super-hydrophilic and super-hydrophobic) are among the most interesting methods for use in solar panel cleaning applications. The self-cleaning surface acts as an anti-dust coating and reduces the accumulation of dust particles^15,16^. Several research groups have been working on anti-reflection and anti-soiling methods for solar panels; however, the coating efficiency tests are always performed in the laboratory. There are few studies that were conducted in real environments, and these are summarized in Table 1. The lowest ΔP was 0.7% achieved from the 1-year test conducted by the Asahi Kasei Corporation in Spain^20^ while the highest ΔP of 11.2% was obtained for the 6-week test conducted by Sketch Co., Ltd. in Egypt, using nano-particles coating together with an automated mechanical vibrator^22^.

**Table 1:** Comparison of average power difference (ΔP) of the coated and uncoated solar panels in real environments.

Materials	Location (duration)	ΔP (%)	References
SurfaShield G (NanoPhos Company)	Attica, Greece (7 months)	5	^17^
SurfaShield G (NanoPhos Company)	Neimeng, China (5 months)	6	^17^
Automatic sprayers and wipers	Greater Noida, India (7 days)	1.8	^18^
Nanopatterned superhydrophilic glass	Singapore (12 weeks)	6.4	^19^
(Unknown) Asahi Kasei Corporation	Malaga, Spain (1 year)	0.7	^20^
Nano-TiO_2_ (ZIXILAI Co. Ltd)	Yverdon-les-Bains, Switzerland (4 days/spray muddy water)	8	^21^
Nano-particles coating (Sketch Co. Ltd.) together with an automated mechanical vibrator	Cairo, Egypt (6 weeks)	11.2	^22^
TiO_2_ nanoparticles (this work)	Chiang Mai, Thailand (10 months)	6.6	This work

Films prepared by the sparking process always have fluffy morphology due to the irregular stacking of primary nanoparticles. This nanostructure results in superhydrophilic properties. Moreover, in this study, titanium wires were chosen as sparking wires in the sparking discharge process to produce TiO_2_ films because of their transparent and photocatalytic properties. The photocatalytic property helps to decompose the organic compounds adsorbed on the surface. Subsequently, contaminants and dust can be easily washed off by rainwater.

## Experimental

### Film preparations

**Figure 1:**
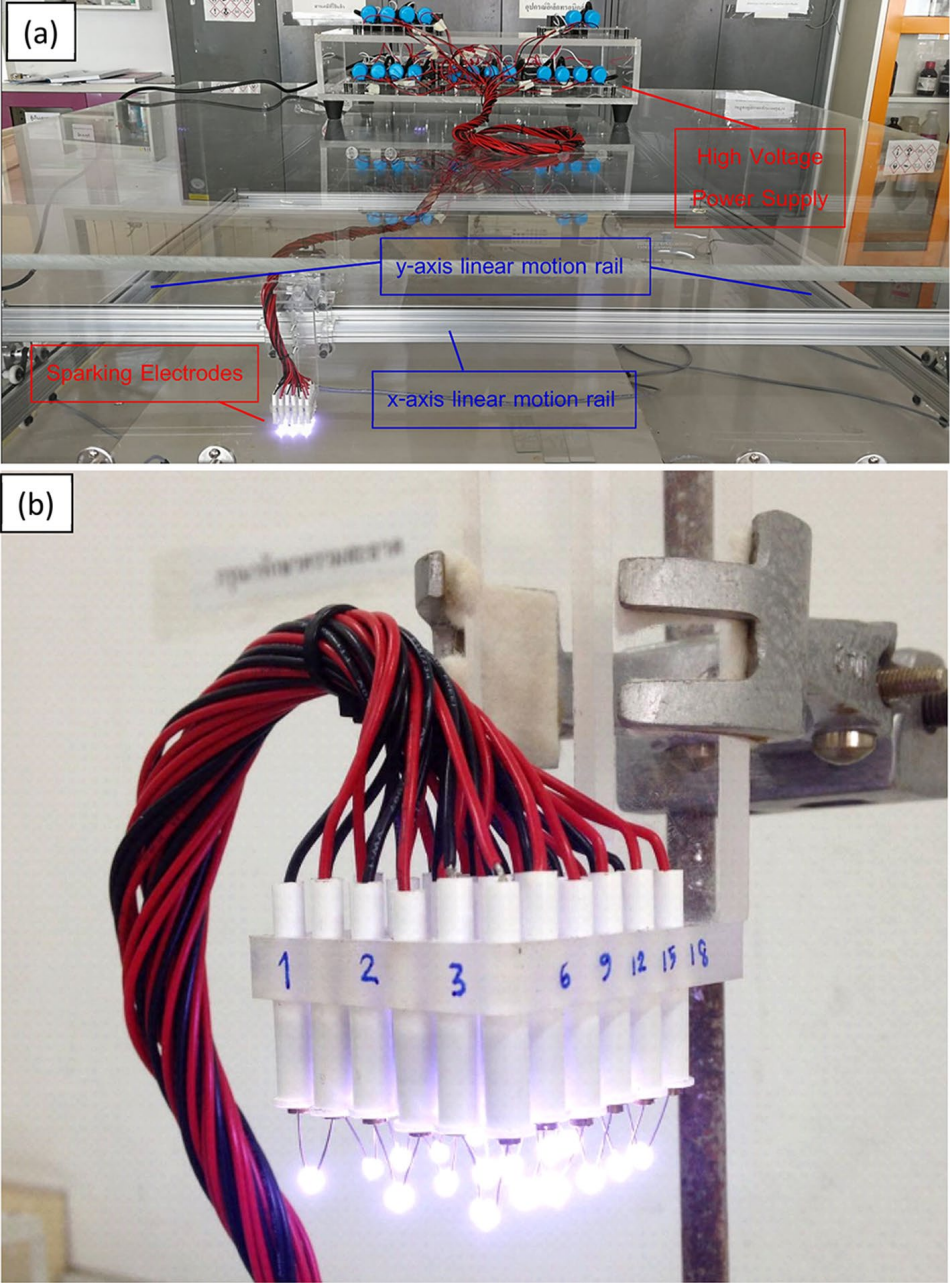
(**a**) Sparking process coating equipment consist of high voltage power supply, x–y axis motion real and sparking electrode (**b**) 18 pairs of Sparking tips.

The sparking instrument used for the fabrication of the titanium nanoparticle films is shown in Fig. 1a. The sparking machine was equipped with six rows and three columns (18 pairs, Fig. 1b, of titanium electrodes that could be moved along two dimensions while the substrate was fixed at 5 mm under the sparking tips. The sparking tips move over the substrates during the coating process, and the speed was fixed at 1 mm/s for this experiment. The design of the sparking system allowed the rapid deposition of nanoparticle films over a large area, especially over stationary substrates such as solar panel systems. Titanium wires (Ø 0.25 mm, purity 99.5%, Advent Research Materials Ltd) were used as the anode and cathode electrodes. To form sparking tips, the wires were cut and aligned with a gap of 1 mm between the anode and cathode. The titanium wires were sparked off with a high DC voltage of ~ 3 kV discharged from a 24-nF capacitor.

**Figure 2:**
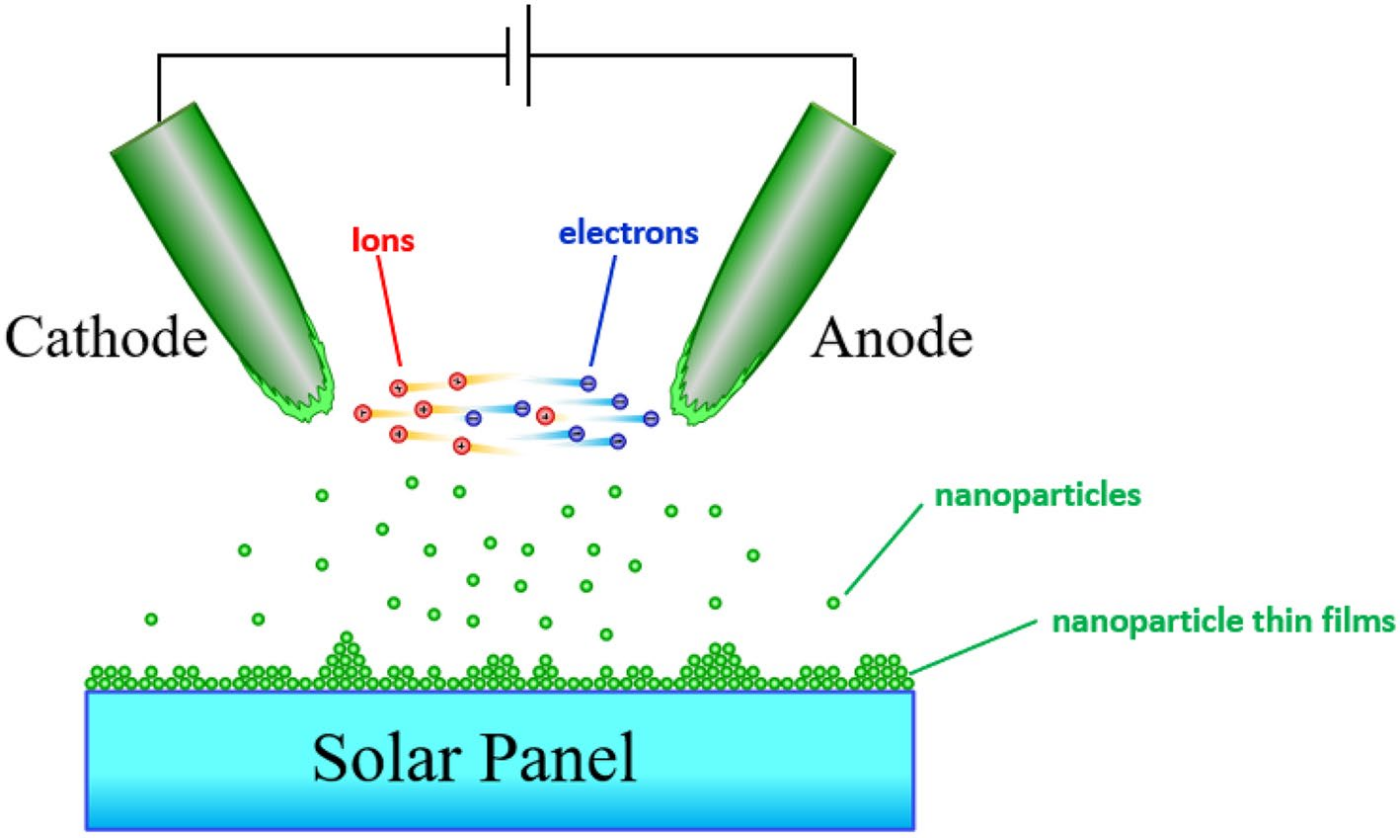
Schematic diagram of the coating mechanism of nanoparticulate films prepared by the sparking process.

The high applied voltage induced arcing in the tip gap via a field ionization process. During sparking, electrons and ions produced from the neutral air molecules migrate toward the anode and cathode, respectively. The bombardment of high-energy electrons and ions melt the metal tips. Hence, the nanodroplets are nucleated, which move toward the substrate and oxidized in atmospheric air. From our previous work^9,11^, the sparked titanium films were anatase TiO_2_. A schematic of the nucleation and growth of the nanostructures is shown in Fig. 2. The arcing electrode was repeatedly scanned over the substrates and the agglomerates of the deposited nanoparticles exhibited a fluffy film structure on the substrate.

### Characterizations

The surface morphology and film thickness were characterized using scanning electron microscopy (SEM, JEOL JSM 6335F). The water contact angles (WCA) were measured using a custom-made tensiometer. The optical transmittance was measured in the range of 350–800 nm using a UV–Vis spectrophotometer (Hitachi U-4100). The photocatalytic properties were investigated by measuring the decomposition of 0.01 mM methylene blue (MB) solution samples after they were placed in a sunbath for 1 h. The surface morphology and roughness were characterized by an atomic force microscope in the tapping mode (AFM, Digital Instruments, Inc., Santa Barbara, CA) equipped with a standard Si tip and operated at a scan size of 10 × 10 µm^2^ in air at room temperature. Due to the limitation of PV cover glass thickness and transparency, the experiments were performed on both the cover and slide glass substrates. The cover glass was used for the WCA, reflectance and AFM measurements whereas slide glass substrates were used for SEM, photocatalytic and transmittance.

### Power generation performance

**Figure 3:**
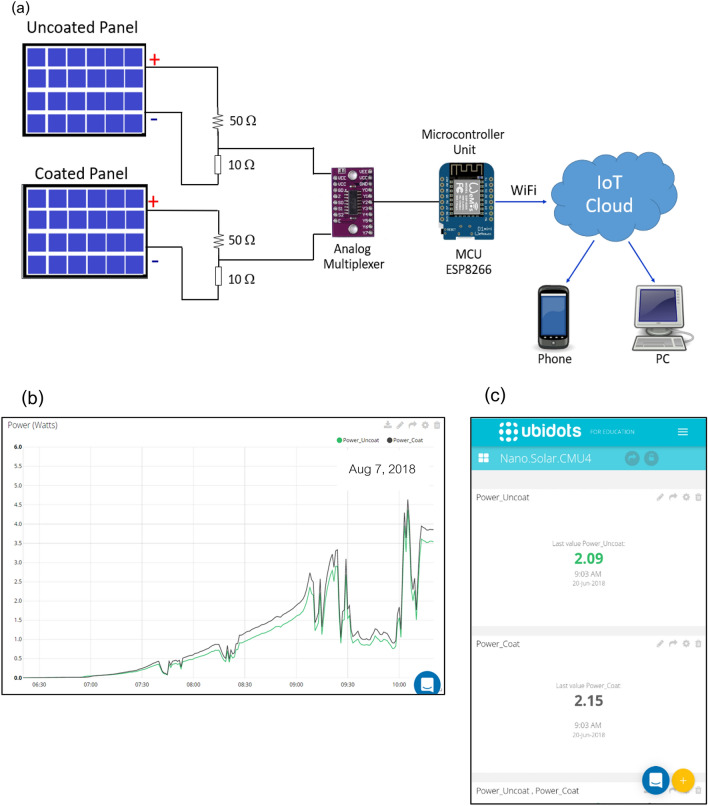
(**a**) Experimental set-up of the panels (**b**) screenshot of Ubidots website (**c**) power generated monitored via Ubidots Explorer application.

As depicted in Fig. 3a, to compare the power generation performance, the coated and uncoated solar panels were exposed to solar irradiation at an inclination of 19° (north–south direction) under prevailing ambient conditions at the Faculty of Science, Chiang Mai University (18° 48′ N, 98° 57′ E), Chiang Mai, Thailand from June 2018 to March 2019. Eight monocrystalline solar panels were used in this experiment, manufactured by Sun Solar Ecotech. The panel width and length are 193 mm and 245 mm, respectively, with a maximum power P_max_ 5 W (± 5%), open circuit voltage V_oc_ 21.5 V, short circuit current I_sc_ 0.32 A, maximum power voltage V_mp_ 17.5 V, and maximum power current Imp 0.29 A. Four samples each of brand new and nanoparticles coated were exposed to the sun in natural weathering. Each panel was directly connected to two identical resistors of 25 W 50 Ω and 25 W 10 Ω as used for a load and two-resistor divider. The voltages across each resistor are connected to the analogue multiplexer and the microcontroller unit (MCU, ESP8266, wemos d1 mini). The microcontroller calculates the electrical power generated by each panel and sends it to the IoT server via a Wi-Fi network (Ubidots for Education platform) every minute. As an illustration, screenshots of the ubidots website from 6:30 to 11:00 a.m. on 7 August 2018 are shown in Fig. 3b. The green and black lines represent the power obtained from the uncoated and coated panels, respectively. In addition, the power generated from both panels can be monitored on a mobile phone via the Ubidots Explorer application, as shown in Fig. 3c.

## Results and discussion

**Figure 4:**
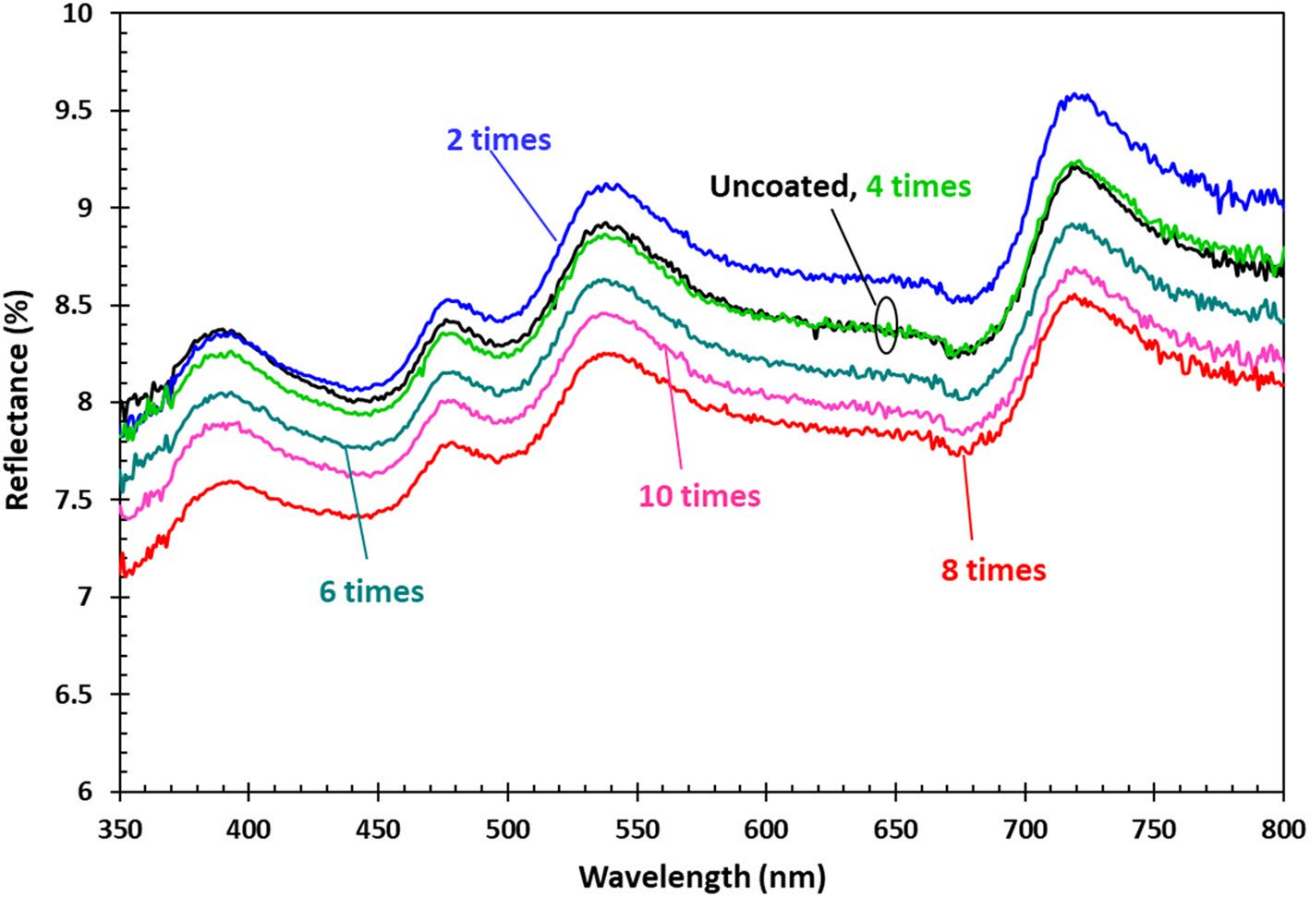
(**a**) Reflectance of the uncoated and the coated sparking titanium on the glass cover of the PV panels. (**b**) Transmittance of uncoated and coated specimen. The inset shows a magnification of the data.

Reflected light represents uncaptured energy; therefore, decreasing the proportion of reflected light represents a promising approach for increasing the efficiency of PV panels. Textures on the front surfaces of the panels are often used to reduce the reflectance; however, it will be significant if the surfaces achieve lower reflective light. To quantify the reflectance of the cover, it was removed from the commercial panels and then cut to a size of 1 × 2 cm^2^, and reflectance measurements were performed. Figure 4 shows the spectral reflectance of the uncoated and coated PV cover glass substrates. Experiments were repeated 3 times with similar results. The results show that the highest reflectance (8–9.5% in visible region) was obtained when the scan was repeated for two cycles. It decreases when the number of scan cycles are increased and the lowest reflectance (7–8.5% in visible region) is obtained for eight cycles. It is interesting to note that the uncoated surface and the surface obtained after four repeated scans have the same level of reflectance.

**Figure 5:**
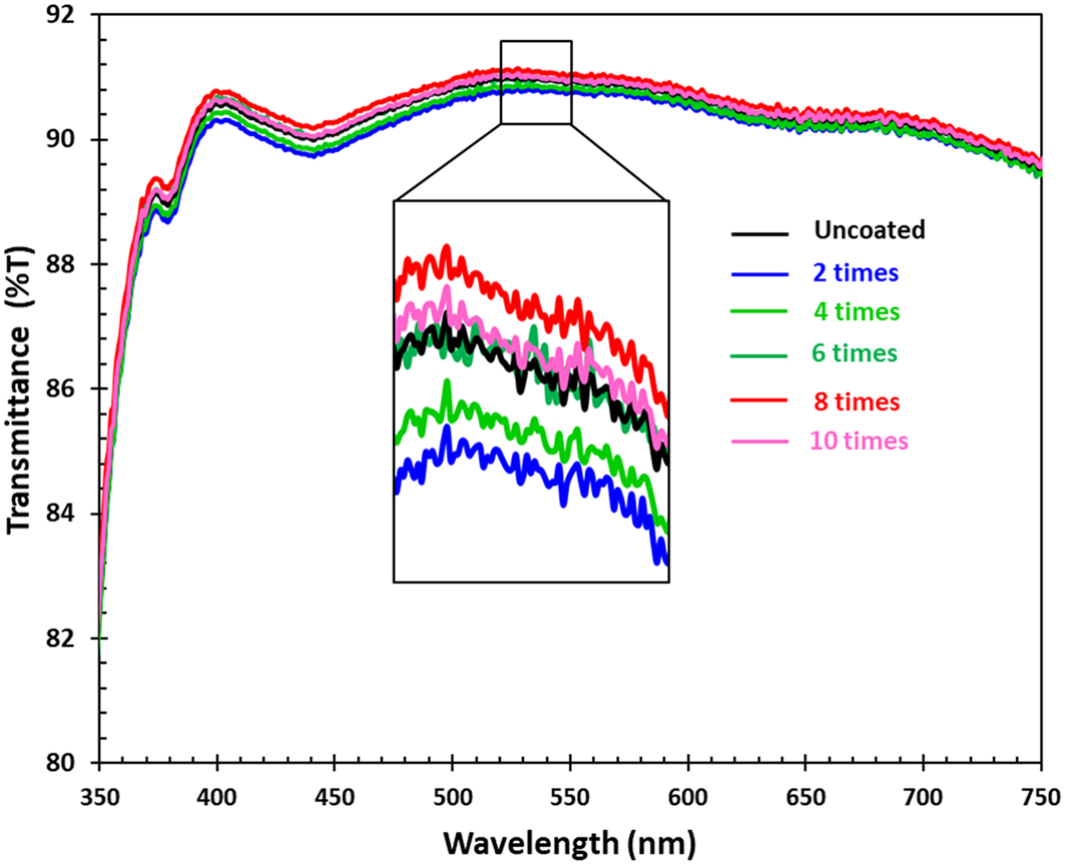
Transmittance of uncoated and coated specimen. The inset shows a magnification of the data.

Figure 5 shows the direct measurement of the optical transmittance spectra of the coated and uncoated films on the glass slides as a function of wavelength. The optical transmittances in the visible region of all the samples were in the range of 90–91%. The repeated scan for two cycles revealed the lowest transmittance, as shown in the inset. After that, the transmittance increased with the increase in repeated scan times and maximum at the repeated scan eight times.

**Figure 6:**
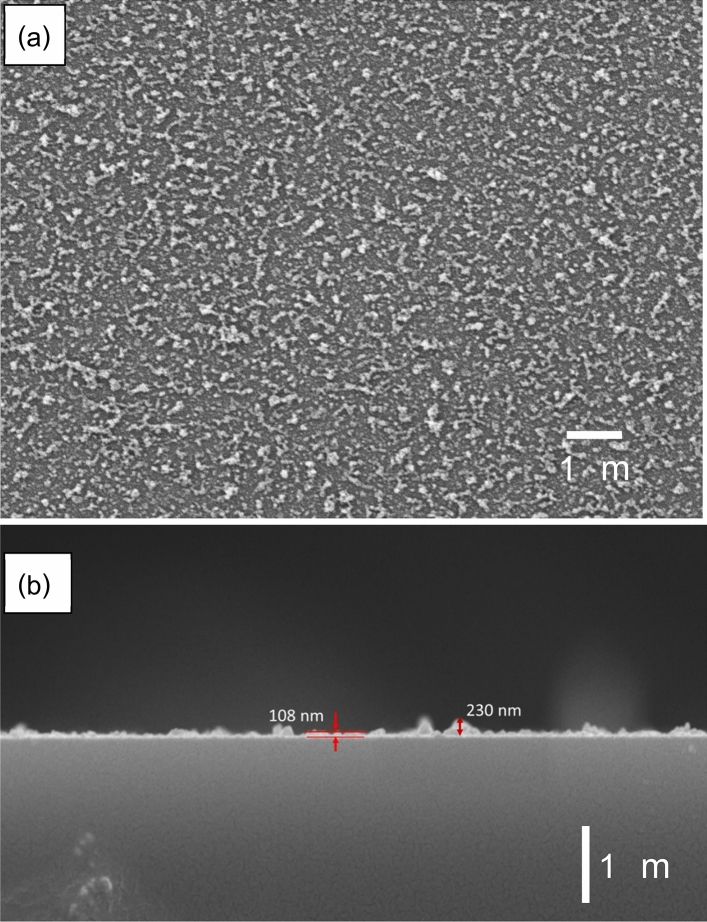
SEM images of top view (**a**) and cross-section (**b**) of glass slide submitted to eight scans.

Figure 6a,b show the top view and cross-section SEM images, respectively, of the eight repeated scans sample. As shown in Fig. 5a, the films have a fluffy morphology due to an irregular stacking of primary nanoparticles, the agglomeration mechanism was explained in our previous report^9^. The cross section of the surface formed on slide glass substrate were examined to determine the thickness of the film (Fig. 6b). It was seen that the nanoparticle films covered the substrate, and some of the particles formed island structures. The thickness of the films was in the range of 108–230 nm.

**Figure 7:**
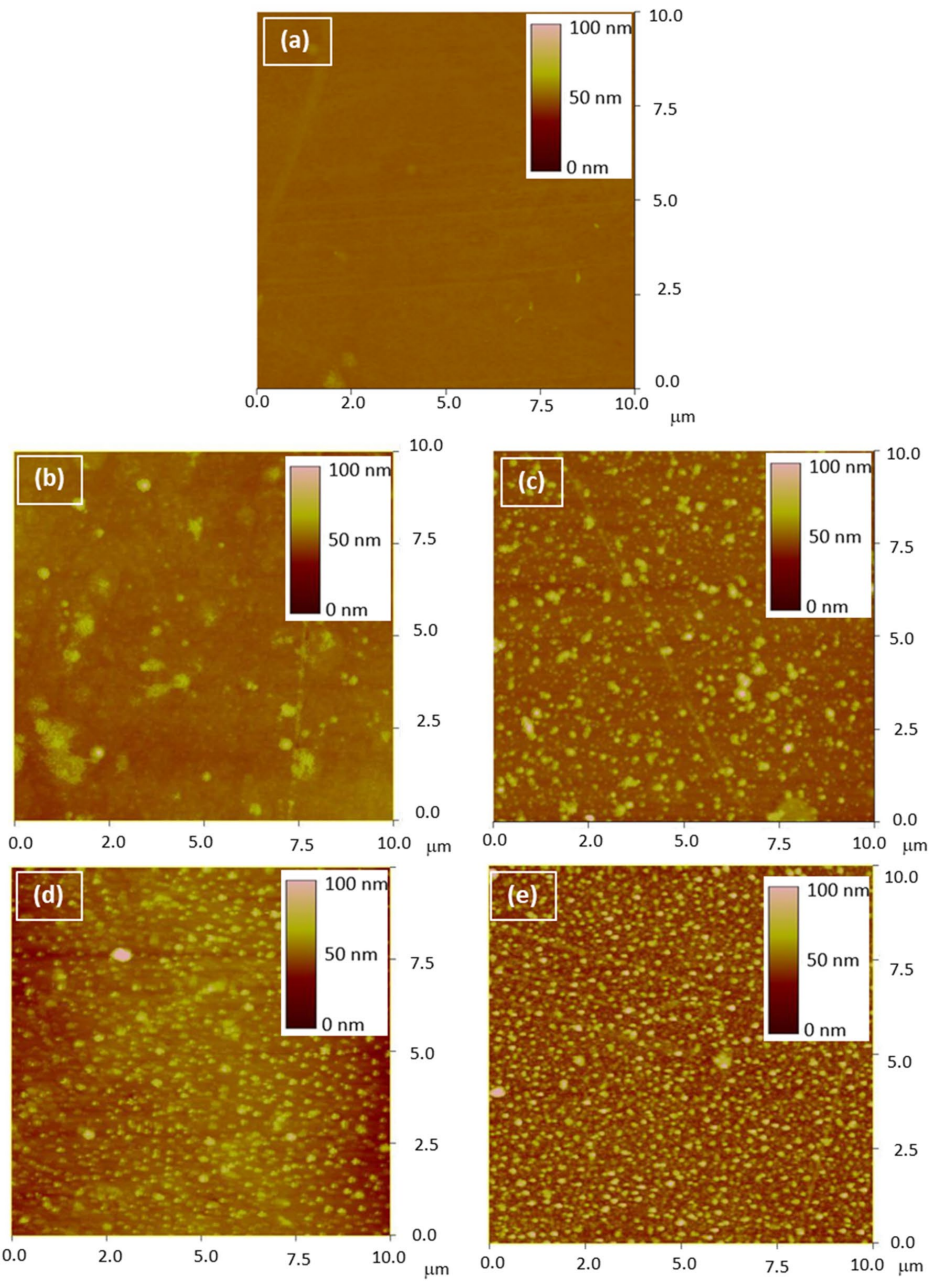
AFM images of the uncoated specimen (**a**) and specimens subjected to 2, 4, 6, and 8 scans (**b**–**e**), respectively.

The surface topography of the uncoated and coated glasses was examined using AFM, as illustrated in Fig. 7. The uncoated sample surface was quite flat with surface roughness values in the vicinity of 2.5 nm and 39.9 nm for R_q_ and R_max_, respectively (Fig. 7a). For the coated substrates, the area covered by the nanoparticles increased with an increasing number of scans. As shown, the AFM image after two scan cycles shown in Fig. 7b indicate the presence of a few nanoparticles, and the roughness values increased to 3.75 nm and 80.4 nm respectively, for R_q_ and R_max_. The film partially covers the surface of specimen subjected to four scan cycles (Fig. 7c), mostly covers the specimen subjected to six scan cycles (Fig. 7d), and covers completely the specimen subjected to eight scan cycles (Fig. 7e). The R_q_ were 6.64, 8.03, and 11.28 nm, while the R_max_ were 130.7 nm, 114.8 nm, and 124.7 nm corresponding to the 4, 6, and 8 scan cycles, respectively.

**Figure 8:**
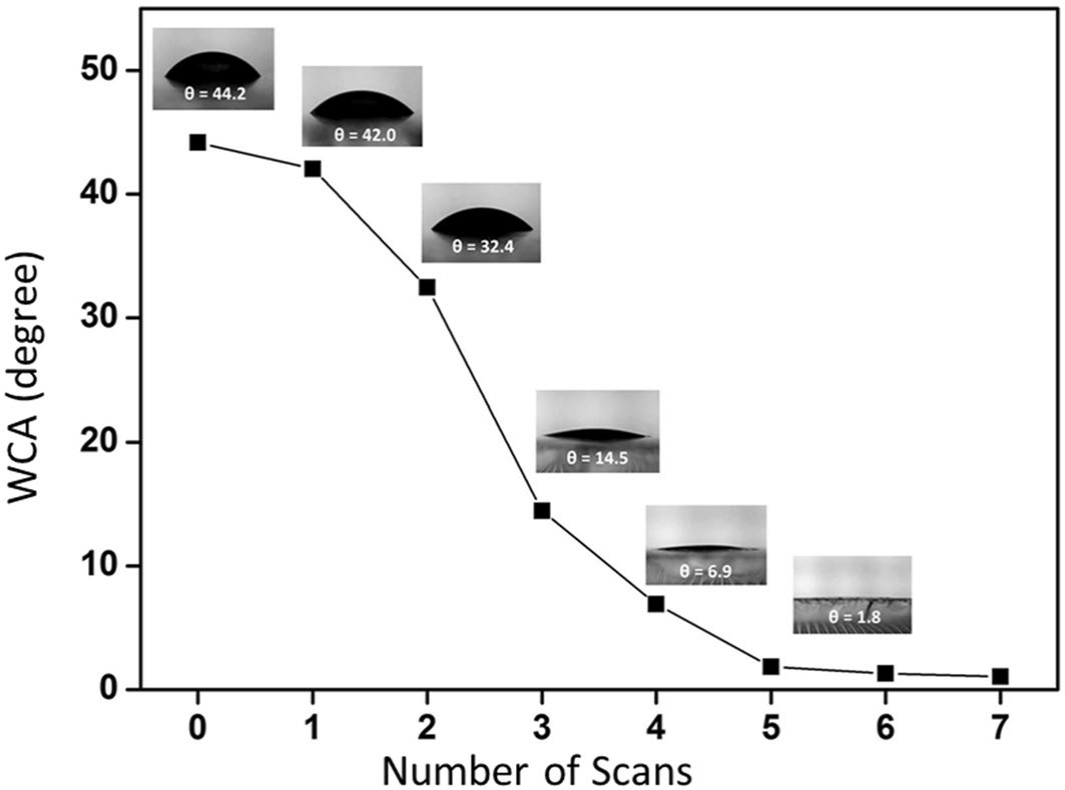
Variation of water contact angle with number of scans.

The static contact angles of a water droplet on the uncoated and coated PV cover glass were measured to determine their hydrophilicity (Fig. 8). All water contact angles (WCA) were determined as averages of three different drops at different positions on the uncoated and coated surfaces. The largest water contact angle was 44.2 ± 1.5° on the glass surface. After the samples were coated with the nanoparticles, the WCA decreased with an increase in the number of repeated scan cycles. The average WCA was 42.0 ± 2.1°, 32.4 ± 1.0°, 14.5 ± 0.5°, 6.9 ± 0.3°, and 1.8 ± 0.2°, respectively, for scans that were repeated 1–5 times. The decrease in the WCA occurs because the nanoparticle films can increase the contact area between the water droplet and substrate and thereby reduce the contact angle^12^. The water droplets were completely flat when the scans were repeated 6–10 times, and the WCA was close to 0° (pictures not shown). This result shows that the surfaces assumed super-hydrophilic states (WCA < 5°) after repeated scanning for five cycles.

**Figure 9:**
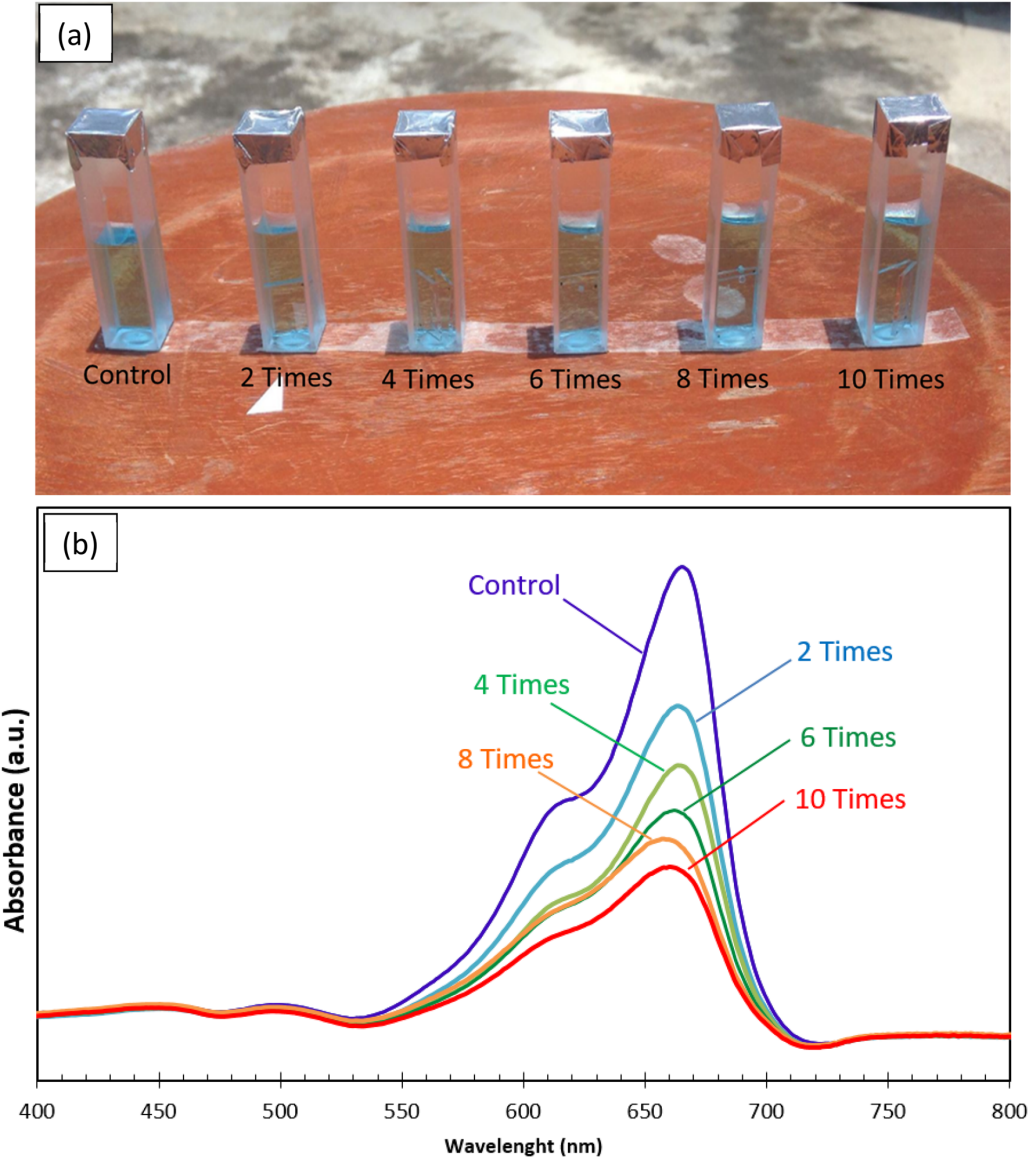
(**a**) photographs of the methylene blues after exposed in sunlight for 1 h (**b**) Variation of absorbance spectrum of methylene blue with wavelength.

To study the photocatalytic properties, the uncoated and coated glass slides 10 × 10 mm^2^ in size were soaked in the 2.5 cm^3^ methylene blue (MB) solution having an initial concentration of 0.01 mM (Fig. 9a). The degradation of MB under sunlight for 1 h at noon was measured to determine the catalytic activity. Figure 9b shows the variation in the absorbance spectra of MB with the wavelength of different repeatedly scanned cycles. The absorption peaks were 665 nm, and the peaks decreased gradually with the increase in the number of scan cycles. In other words, the degradation of MB increased gradually with the number of scan cycles. The degradation of MB indicates that the coatings may exhibit self-cleaning activity for other organic contaminants on the cover surface of PV panels and hence, increased efficiency of solar light utilization.

**Figure 10:**
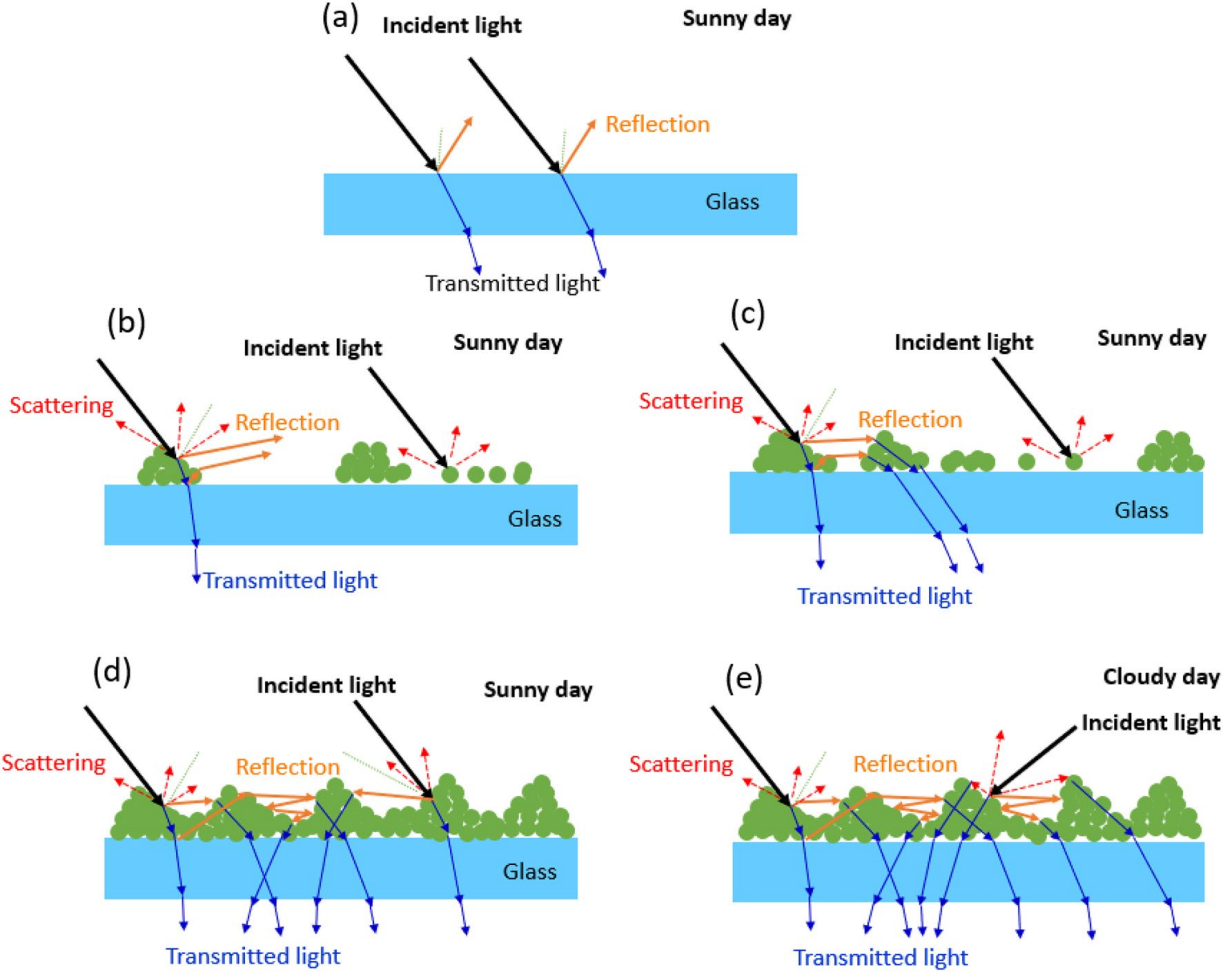
Schematic diagram represent the light interaction on sunny days with (**a**) glass surfaces (**b**) surface subjected to two scans (**c**) surface subjected to four to six scans (**d**) surface subjected to eight scans. (**e**) Surface subjected of eight scans on a cloudy day.

In our previous reports^9,23^, the distribution of nucleated nanoparticles on the substrates are explained. They randomly distributed on a substrate and some particles attached to others and created a secondary particle. Therefore, the substrate surfaces were better coverage for more repeated scan times. Figure 10a–e shows a schematic of the cross section and the behavior of the incident light on the solar panel glass. For an uncoated surface, light is reflected from the front surface (Fig. 10a). For the specimen subjected to two scan cycles, as shown in Fig. 10b, the incident light scattering caused by the nanoparticles and their stacks caused an increase in the reflective light. When the number of scan cycles was increased to four and six cycles, the reflectance decreased because the island structures could trap some reflective light, but the isolated nanoparticles still caused scattering (Fig. 10c). The nanoparticles having an island structure cover the entire surface for eight cycles as shown in Fig. 10d. This film structure has the highest light-trapping efficiency compared to a lower number of repeated scan cycles. Moreover, on a cloudy day, the clouds scatter light, making it move along different directions at the panel surfaces. As shown in Fig. 10e, the same film structure traps more scattered light compared to direct light on sunny days; hence, the power difference on the cloudy day is much more than that on a sunny day.

**Figure 11:**
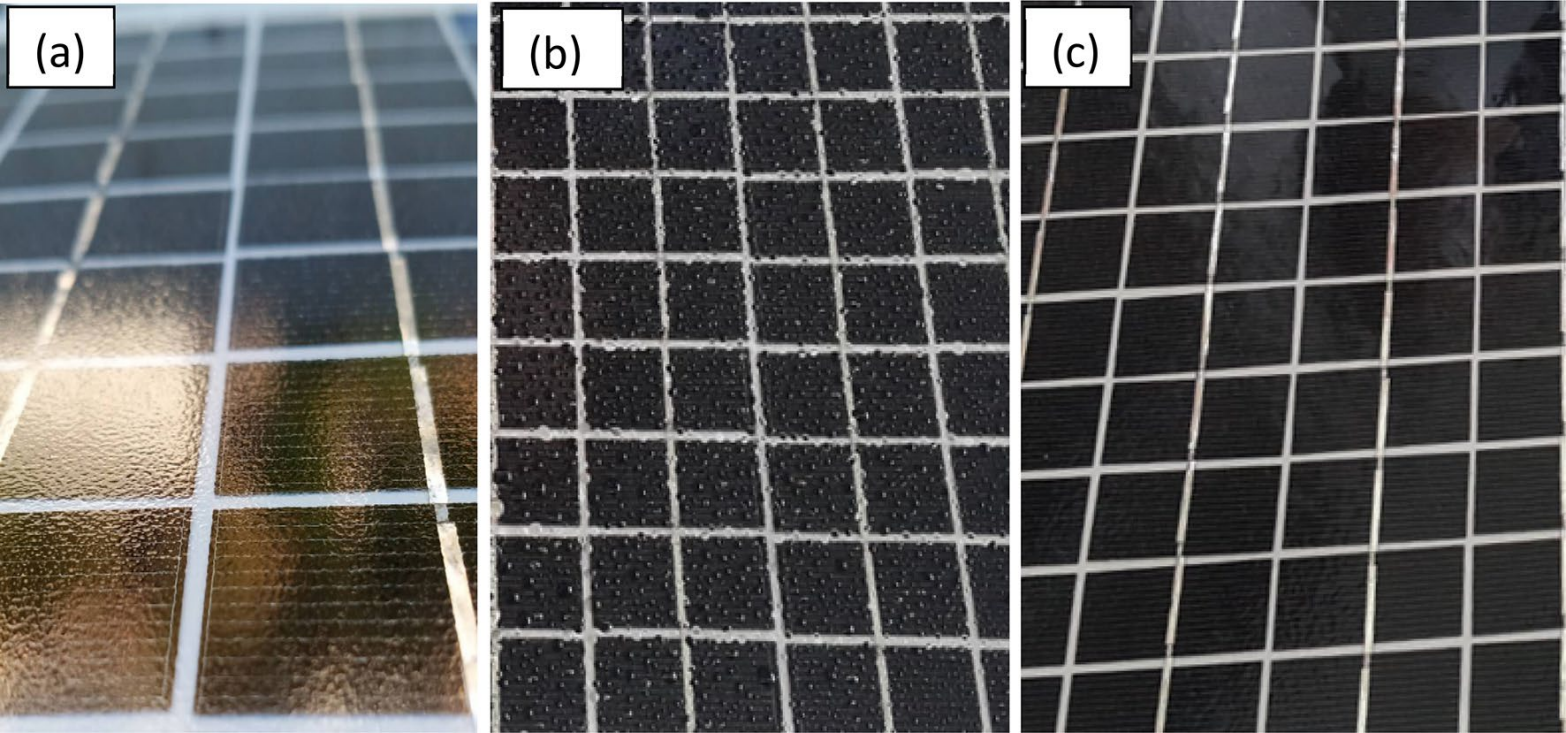
(**a**) Uncoated surface of PV panels (**b**) and (**c**) surface of uncoated and coated PV panels, respectively, after water spraying.

One of the most important issues that limit the efficiency of solar cells is soiling. Drizzle-type rainfall enhanced dust adhesion on the panels, which converted this dust into a muddy stain that was difficult to remove without cleaning. Superhydrophilic surfaces are anti-soiling coatings that allow water to spread across the substrate and carry away dirt, rather than form droplets that leave stains on top of the substrates after drying^24^. Due to the lowest light reflectance and superhydrophilicity of the repeatedly scan for eight cycles, this experimental condition was selected to prepare the nanoparticles films on the solar panels. Figure 11 shows the wetting of the coated and uncoated panels. The uncoated cover glass of the panel is shown in Fig. 11a, and the water droplets remaining on the surface after spraying with water are shown in Fig. 11b. The droplets caused the formation of muddy stains after drying and resulted in a reduction in the power generated by the panels. In contrast with the uncoated panels, there were no water droplets on the coated panel after spraying with water (shown in Fig. 11c).

**Figure 12:**
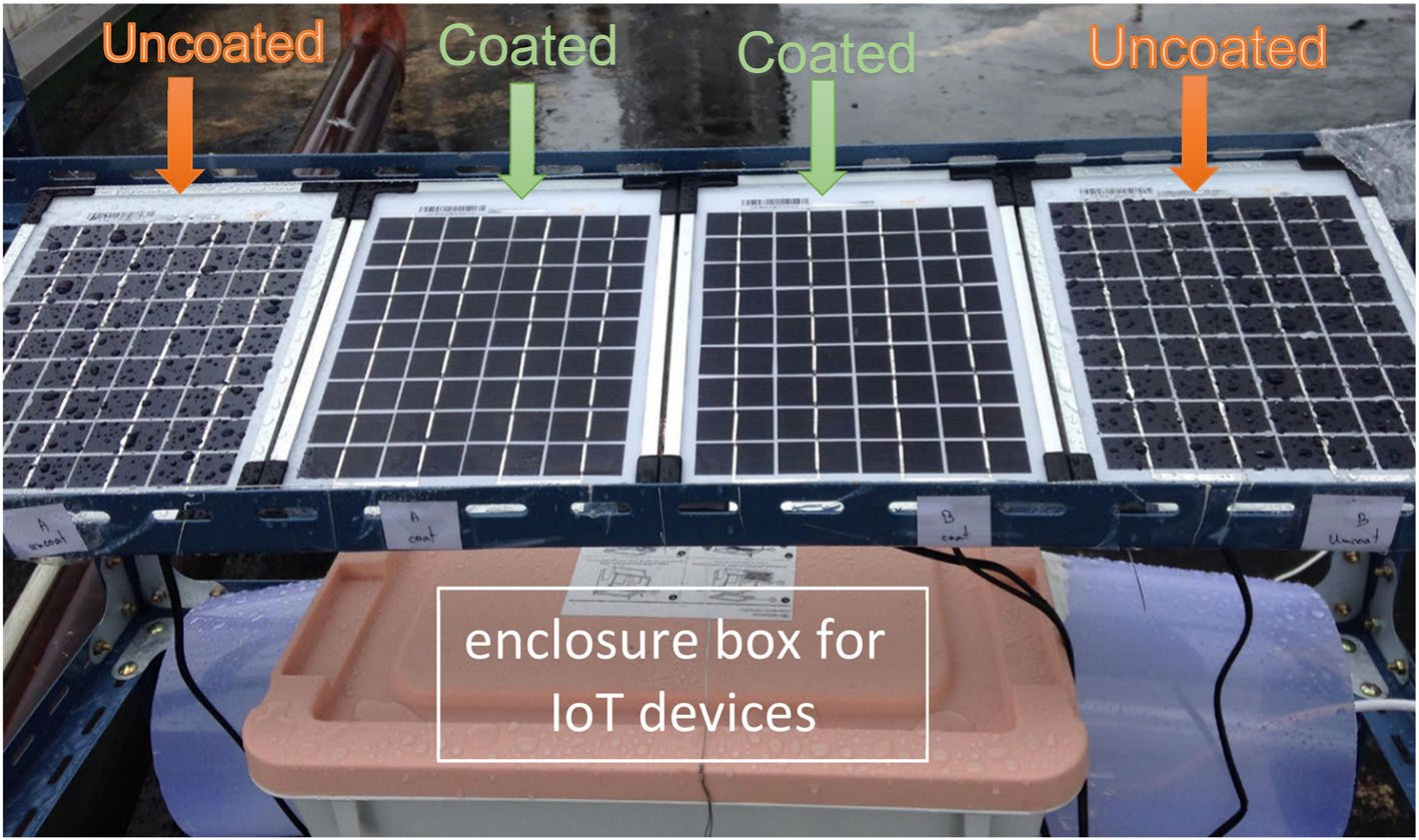
Picture of the uncoated and coated PV panels after rain.

The outdoor experimental station was located at the Faculty of Science, Chiang Mai University (18.801468, 98.956036); the panels used here faced south and the tilt of the panels was 19°. Four uncoated and four coated samples were used in this study. As shown in Fig. 12, the coated panels were placed in the middle and the uncoated panels were placed on the edges. The behavior of rainwater droplets on the coated and uncoated surfaces are illustrated in the figure. The results are the same as those shown in Fig. 11, the rainwater spread across the coated panels whereas the droplets were formed on the uncoated surfaces.

**Figure 13:**
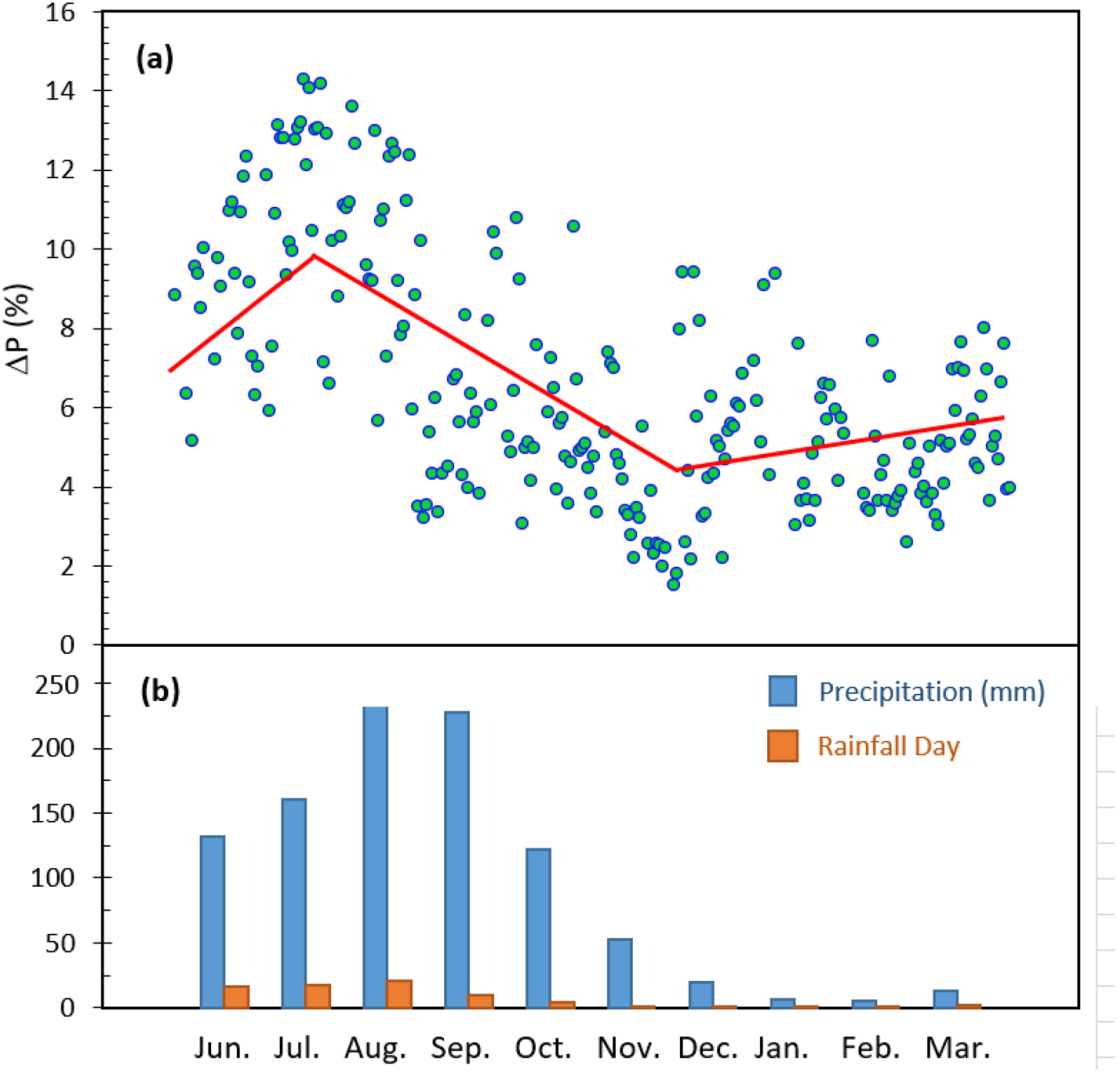
(**a**) The differences in the average daily output power (**b**) precipitation and rain fall days in the experimental period.

The differences in the average daily output power and the output power of the coated and uncoated samples from 7:00 a.m. to 5:00 p.m. on each day during the period from June 2018 to March are shown in Fig. 13a. Considering that all coated and uncoated panels were still clean, the average power difference per day (ΔP) was 5–9% due to the entrapment of scattered light on cloudy days. ΔP tended to increase during the first three months and the highest value of 14.22% was recorded on 31 July 2018. The increase in ΔP is due to two reasons. Firstly, rainy days are associated with cloudy skies with more scattered light, and so, the coating can produce more power by light entrapment. Secondly, during the early rainy season in Thailand, drizzles increased dust accumulation on the panels. Muddy stains were observed on the uncoated panels after the rain. The coating was super-hydrophilic; therefore, there was less dust than the uncoated panels. In August–October, there was enough rain to clean all panels, and there were fewer cloudy days resulting in a decreasing trend in ΔP. Finally, ΔP exhibited steady activity during December 2018–March 2019. This is associated with the dry season. The precipitation and rainfall data in Chiang Mai pertaining to the period of the study are also shown in Fig. 13b (data from https://www.chiang-mai.climatemps.com/precipitation.php). Overall, the data collected revealed an average power gain of 6.62% due to the coating during the entire study period.

**Figure 14:**
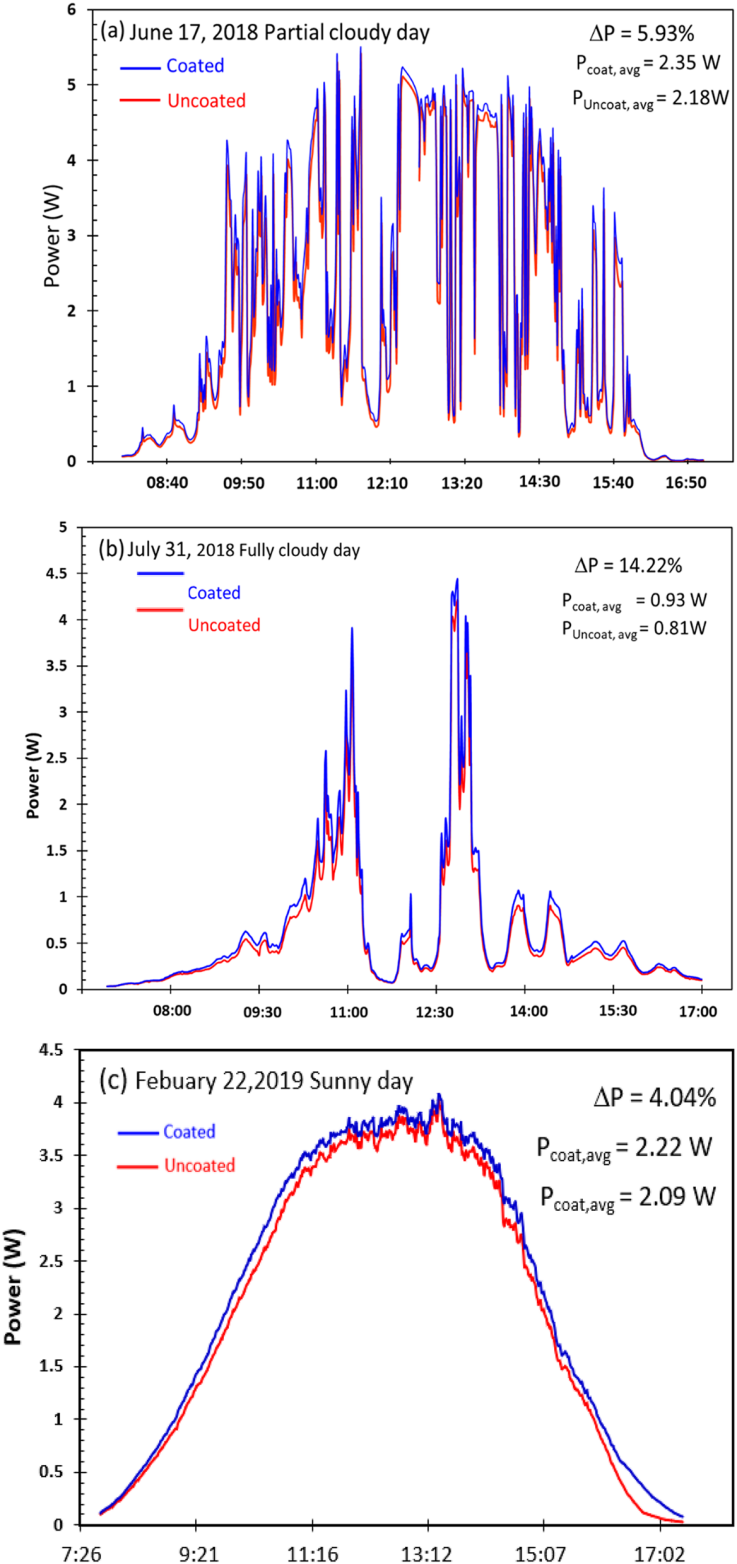
ΔP measured during (**a**) partially cloudy day (**b**) rainy day and (**c**) sunny day.

To study the characteristic of power difference in the partially cloudy day, the rainy day and the sunny day, the power generated in three difference days were considered as shown in Fig. 14. The power generated by the coated and uncoated surfaces on the third day of the experiment (17 June 2018) is shown in Fig. 14a. It was a partially cloudy day with intervals of sunlight, and the plot for both power values are quite irregular due to the solar irradiance drop. The value of ΔP was recorded as 5.38%. On the 31st of July, which was a rainy and cloudy day with little sunlight interval, the power plots lay below 2 W throughout the day. The plot spiked twice at approximately 11:00 and 13:00, indicating that the sun had appeared. On this day, ΔP reached the highest recorded value of 14.22%, as shown in Fig. 14b. This is because the cloudy days have a higher component of scattered light; therefore, the coated panels trapped light using the nanoscale particles that form the nanoparticle films. Figure 14c shows the power generated on 22 February 2019. It is a sunny day, and the plots of the power generated by the coated and uncoated panels resemble a parabola opening downward. The ΔP was 4.04%, which was less than that for partially cloudy and rainy days.

## Conclusion

In this study, we introduced an inexpensive coating fabricated via the sparking of titanium wires that can improve the power conversion efficiency of solar panels. The coated films demonstrated excellent anti-reflective, super-hydrophilic, and photocatalytic properties. The long-term outdoor experiment demonstrated that the power difference per day (ΔP) increased significantly owing to the reduction of dust accumulation on the panel surface. ΔP reached the highest value of 14.22% in scattered light (cloudy day), which may be attributed to the antireflective properties of the coating. Over the entire period, the power output of the coated panels was 6.62% higher than that of the uncoated panels.
